# Performance of Different Analytical Software Packages in Quantification of DNA Methylation by Pyrosequencing

**DOI:** 10.1371/journal.pone.0150483

**Published:** 2016-03-02

**Authors:** Chiara Grasso, Morena Trevisan, Valentina Fiano, Valentina Tarallo, Laura De Marco, Carlotta Sacerdote, Lorenzo Richiardi, Franco Merletti, Anna Gillio-Tos

**Affiliations:** 1 Cancer Epidemiology Unit – C.E.R.M.S, Department of Medical Sciences, University of Turin, Turin, Italy; 2 Cancer Epidemiology Unit, Department of Medical Sciences, City of Health and Science Hospital, Turin, Italy; Sapporo Medical University, JAPAN

## Abstract

**Background:**

Pyrosequencing has emerged as an alternative method of nucleic acid sequencing, well suited for many applications which aim to characterize single nucleotide polymorphisms, mutations, microbial types and CpG methylation in the target DNA. The commercially available pyrosequencing systems can harbor two different types of software which allow analysis in AQ or CpG mode, respectively, both widely employed for DNA methylation analysis.

**Objective:**

Aim of the study was to assess the performance for DNA methylation analysis at CpG sites of the two pyrosequencing software which allow analysis in AQ or CpG mode, respectively. Despite CpG mode having been specifically generated for CpG methylation quantification, many investigations on this topic have been carried out with AQ mode. As proof of equivalent performance of the two software for this type of analysis is not available, the focus of this paper was to evaluate if the two modes currently used for CpG methylation assessment by pyrosequencing may give overlapping results.

**Methods:**

We compared the performance of the two software in quantifying DNA methylation in the promoter of selected genes (GSTP1, MGMT, LINE-1) by testing two case series which include DNA from paraffin embedded prostate cancer tissues (PC study, N = 36) and DNA from blood fractions of healthy people (DD study, N = 28), respectively.

**Results:**

We found discrepancy in the two pyrosequencing software-based quality assignment of DNA methylation assays. Compared to the software for analysis in the AQ mode, less permissive criteria are supported by the Pyro Q-CpG software, which enables analysis in CpG mode. CpG mode warns the operators about potential unsatisfactory performance of the assay and ensures a more accurate quantitative evaluation of DNA methylation at CpG sites.

**Conclusion:**

The implementation of CpG mode is strongly advisable in order to improve the reliability of the methylation analysis results achievable by pyrosequencing.

## Introduction

DNA methylation is an epigenetic modification involved in the regulation of several biological processes. Methylation of nucleotide bases may lead to N6-methyladenine (6mA), N4-methylcytosine (4mC), and 5-methylcytosine (5mC). While 6mA and 4mC are restricted to prokaryotes and certain eukaryotes, 5mC is the predominant epigenetic modification in eukaryotic DNA.

In healthy mature mammalian cells DNA methylation mainly involves a Cytosine when it is followed by a Guanine (CpG dinucleotides or CpG sites) [1–3]. Most of the mammalian genes contain CpGs clustered in short regions called CpG Islands and located in the promoter, where methylation may drive silencing of the gene. Non-CpG methylation may occur within some immature type of cells (stem cells) at specific stages during development or in some cell tissues at low proliferative rate (brain, oocytes) [4]. Its functional significance in the mammalian genome is poorly understood and the mechanism is studied at a lesser extent. On the contrary dysregulations in the physiological process of CpG methylation are known leading to abnormal silencing or activation of genes, with potential alteration in cell cycle control and disease onset [3]. The most relevant pathological outcome of aberrant CpG methylation is cancer, thus many efforts have been made in recent decades to identify genes affected by aberrant DNA methylation at CpG sites associated with early carcinogenesis. The analysis of human gene-specific DNA methylation can be performed under a variety of molecular protocols following sodium bisulfite modification of the genomic DNA. Sodium bisulfite treatment deaminates the unmethylated cytosines to uracil whereas leaves unaltered the methylated cytosines [5]. Therefore methylated and unmethylated DNA sequences become distinguishable using primer/probe specific PCR, microarray or sequencing mediated methodologies. Several of these methods are expensive, time consuming, not quantitative, or limited to the measurement of the methylation status of only one or very few CpG sites [6,7].

Pyrosequencing overcomes these limitations and allows the simultaneous analysis of several CpG sites up to 100 bp amplicon length [8, 9]. This method is monitored by bioluminescence and based on DNA sequencing-by-synthesis and luminometric detection of pyrophosphate release through a series of enzymatic reactions [10–12]. After bisulfite modification of the genomic DNA, the region of interest is amplified by PCR employing one of the two primers biotinylated. The amplicon is rendered single stranded and a pyrosequencing primer is annealed to quantitatively analyse the methylation within the CpG sites of the target sequence. Nucleotides are added in a predetermined order in each pyrosequencing cycle. Each incorporated nucleotide event is accompanied by release of pyrophosphate and results in a proportional emission of light. DNA methylation ratios are calculated from the levels of light emitted by each nucleotide incorporated at the individual CpG positions using a dedicated software. The results are displayed as an average methylation level for each CpG assayed across all the amplification products. The methylation detection limit at individual CpG sites is approximately 5% [9].

Pyrosequencing is a suitable methodology for the analysis of short DNA sequences such as those extracted from paraffin-embedded specimens. The pyrosequencing technology benefits of ease of its implementation, quantitative nature of the results, ability to differentially identify methylated positions in close proximity. Moreover a low amount (10 ng) of bisulfite-treated DNA is requested to obtain high reproducibility and to avoid random amplification [13]. Among the commercially available pyrosequencing systems, two models are used for methylation analyses of candidate genes: PyroMark Q96 and PyroMark Q24 (both purchased by Qiagen, Hilden, Germany). They both carry software for analysis in AQ (Allele Quantification) mode, originally generated for SNP and mutational analysis, and they mainly differ for the number of testable samples in a run, 96 or 24 respectively. An integrated software was created for methylation analyses in CpG mode (Pyro Q-CpG^™^ software v. 1.0.9 and upgrades, Qiagen), that in the latter system of more recent generation is present by default, while in the former has to be implemented. However,also the software which allows analysis in AQ (Allele Quantification) mode has been recommended for CpG methylation analysis [14, 15] and has been widely employed for this purpose. Only since 2007 some reports declared the use of the Pyro Q-CpG^™^ software [16–23]. Several investigations on CpG methylation also recently published have been performed with AQ mode instead of CpG mode, although proof of equivalent performance of the two software for this purpose is currently not available.

The present study aims to compare the performance in the quantification of CpG methylation in human samples of the two software which allow analyses in CpG mode and in AQ mode respectively, to evaluate if they may give overlapping results

## Materials and Methods

### Study samples

To compare the performance of the two software for methylation analysis in CpG or in AQ mode onto two PyroMark Q systems two sets of DNA samples were used, already collected in the frame of broader studies with their own specific aims approved by the local Ethical Committee of the San Giovanni Battista Hospital—CTO/CRF/Maria Adelaide Hospital of Turin. Patient record and information were anonymized and de-identified prior to analysis. The first set included 36 DNA samples obtained from paraffin embedded prostate cancer tissues of patients enrolled in a study of association between gene specific methylation and prostate cancer mortality, thereafter named “PC study” [24]. This sample series was tested for methylation status in the promoter of GSTP1 (glutathione S-transferase-pi 1) gene.

The second set included 28 DNA samples obtained from stored buffy coat of healthy voluntaries involved in a study of association between diet and DNA damage in heavy smokers, hereafter named “DD study”[25]. This sample series was tested for methylation status in the promoter of MGMT (O-6-methylguanine-DNA methyltransferase) and LINE-1 (long interspersed nuclear element type-1).

### DNA extraction and sodium bisulfite treatment

For the case series including paraffin embedded tissues (PETs) of “PC study”, genomic DNA was extracted from 3–5 (10 μm thick) sequential sections of PETs through QIAamp DNA FFPE (Qiagen) according to the manufacturer’s instructions and checked for adequacy by PCR amplification of the β-globin gene.

For the case series including stored blood fractions from “DD study”, DNA was obtained from 100 μl aliquots of buffy coat through QIAamp DNA Blood Mini Kit (Qiagen) according to the manufacturer’s instructions.

All genomic DNA samples, as well as synthetic controls for methylated and unmethylated status, underwent bisulfite modification using the Epitect Bisulfite Kit (Qiagen).

### Pyrosequencing

Pyrosequencing assays were performed for all the study samples both on a PyroMark Q24 MDx and on a PyroMarkQ96 ID using PyroMark Gold reagents (Qiagen). Primers for GSTP1, targeting 4 CpGs in the gene promoter, were generated according to PyroMark Assay Design software version 2.0 (Qiagen). Primers used for the assay of LINE-1, targeting 6 CpGs in the gene promoter, were chosen according to the literature [26], as well as those for MGMT, targeting 6 CpGs in the gene promoter [27]. Primer sequences are listed in Table 1.

**Table 1:** Pyrosequencing primer sequences and annealing profile.

Gene	Primer	Primer sequence	PCR annealing T°	Target sequence bp	CpG sites
GSTP1	sense	5’-GATTTGGGAAAGAGGGAAAGGT- 3’	50	72 bp	4
	antisense	Biot-5’-CAAAAAAACGCCCTAAAATCC- 3’			
	sequencing	5’-GGTTTTTTYGGTTAGTTG-3’			
LINE-1	sense	Biot-5’-TAGGGAGTGTTAGATAGTGG-3’	Td* 62→55	108 bp	6
	antisense	5’–AACTCCCTAACCCCTTAC- 3’			
	sequencing	5’-AACTCCCTAACCCCTTAC- 3’			
MGMT	sense	5’- GTATTAGGAGGGGAGAGATT- 3’	Td* 62→59	194 bp	6
	antisense	Biot-5’-CCTTAATTTACCAAATAACCC- 3’			
	sequencing	5’-GGGATTTTTATTAAG- 3’			

* Td: Touch down

PCR reactions were performed in a total volume of 35 μl containing 1X buffer (KCl), 2mM MgCl_2_, 200 μM dNTPs, 0.5 μM of each primer, 1.75U Taq polymerase and 6 μl of bisulfte modified DNA with the following cycling profile: 95°C for 1 min followed by 45 cycles of denaturation at 95°C for 1 min, annealing at the specific temperature for 1 min, extension at 72°C for 1 min. Extension at 72°C for 10 min was finally performed. Amplicons were analyzed by gel electrophoresis on a 2% agarose gel stained with ethidium bromide and visualized by ultraviolet trans-illumination. The residual PCR product (28 μl for AQ mode, 20 μl for CpG mode) was added to distilled water (12 μl for AQ mode, 18 μl for CpG mode) and incubated under shaking with binding buffer pH 7.6 (37 μl for AQ mode, 40 μl for CpG mode) containing 10mM Tris-HCl, 2 M NaCl, 1mM EDTA, and 0.1% Tween 20, added with sepharose beads (3 μl for AQ mode, 2 μl for CpG mode) covered by streptavidin. PCR products were washed with ethanol 70%, denatured with NaOH 0.2 M and re-washed with Tris-Acetate 10 mM pH 7.6. Pyrosequencing reaction was performed for AQ mode in a total of 45 μl, including 44.82 μl of 20 mM Tris-Acetate and 5 mM MgAc_2_, and 0.18 μl of 50 μM sequencing primer (final concentration 0.3 μM); for CpG mode it was performed in a total of 25 μl, including 24.85 μl of 20 mM Tris-Acetate, 5 mM MgAc_2_ and 0.15 μl of 50 μM sequencing primer (final concentration 0.3 μM). Assays were created according to manufacturer’s instruction. The nucleotide dispensation order was outlined by the software Q24 2.0.

### Analytical Software

Pyromark Q96 ID version 1.0.9 software, allowing analysis in AQ mode, was used to generate and automatically analyze pyrograms resulting from sequencing onto PyroMark Q96 ID system.

Pyromark Q24 version 2.0 software, allowing analysis in CpG mode, was used to generate and automatically analyze pyrograms resulting from sequencing onto PyroMark Q24 MDx system.

Quantitative methylation results were considered both as percentage of individual CpG sites and as average of the methylation percentage of all the investigated CpGs. Only the latter is herein reported.

Design of a methylation assay in AQ or in CpG mode mainly differs for two functions which make the CpG mode more stringent than the AQ mode: the first function enables the insertion of bisulfite controls in the sequence to monitor the complete conversion of the non-methylated cytosines. The second function concerns the dispensation order: the two modes include different empty bases as negative controls in specific positions to ensure the accuracy of the methylation assay.

Following quantification of the methylation at each CpG site, the quality of the result at each position is rendered by both the software through a colour-based score: blue, when quantification result is assessed as “passed” therefore acceptable; yellow, when a problem is encountered in the result interpretation, and the result is graded as “to check”; red, when coexisting problems lead to hard interpretation of the pyrogram, and the result is assessed as “failed”.

Colour assignment by the software is based on a series of analytical parameters: a coefficient for height adjustment of the A peak, peaks width, dispensation order, pre-sequencing signal and baseline drift. A non-compliance with these parameters leads to warning messages.

While in the CpG mode always all the parameters are considered, in the AQ mode the last two items are never evaluated by default: they can be optionally included by checking the corresponding box. Moreover, the accepted height of the reference peak is quite different: in the CpG mode a single peak height of more than 20 RLU (relative light units) gives acceptable results (blue score), between 10 and 20 RLU results have to be checked (yellow score), below 10 RLU results are scored red, meaning that the run height is too low to consider the quantification reliable. These parameters can be manually changed by the operator, but they can not be lowered below 5 RLU. In the AQ mode a similar alert appears, “low signal-to-noise ratio”, but it does not display numerical and stringent parameters and it only appears when the quantified signal is too weak to be distinguished from the baseline, that is the signal captured from negative dispensations. Therefore the AQ mode accepts as “passed” runs with very low pick height, also below 5 RLU.

Stringency levels (SL), considering the deviation pattern and the deviation sum in variable positions, are defined in a qualitative way in both modes and set as “normal” by default, but are modifiable by the user in “low” or “high”. When the pyrogram does not satisfy the normal stringency level, the following warning messages can appear: uncertain/failed due to high peak high deviation at dispensation; uncertain/failed due to high sum deviation in variable position; uncertain/failed surrounding reference sequence pattern.

### Statistical analyses

The unweighted kappa statistic were computed by PC-SAS software (version 9.2; SAS Institute, Cary, NC, USA) to determine the level of chance-adjusted agreement between replicates. Kappa values of 0.0–0.2, 0.21–0.40, 0.41–0.60, 0.61–0.80, 0.81–0.99, and 1.0 indicate poor, slight, moderate, substantial, almost excellent, and excellent agreement, respectively [28].

Agreement was also assessed using plots according to Bland-Altman [29] and Pitman’s test of difference in variance (STATA 11.1, StataCorp LP, College Station, TX).

## Results

### GSTP1 methylation analysis employing PyroMark CpG and AQ mode

**Fig 1:**
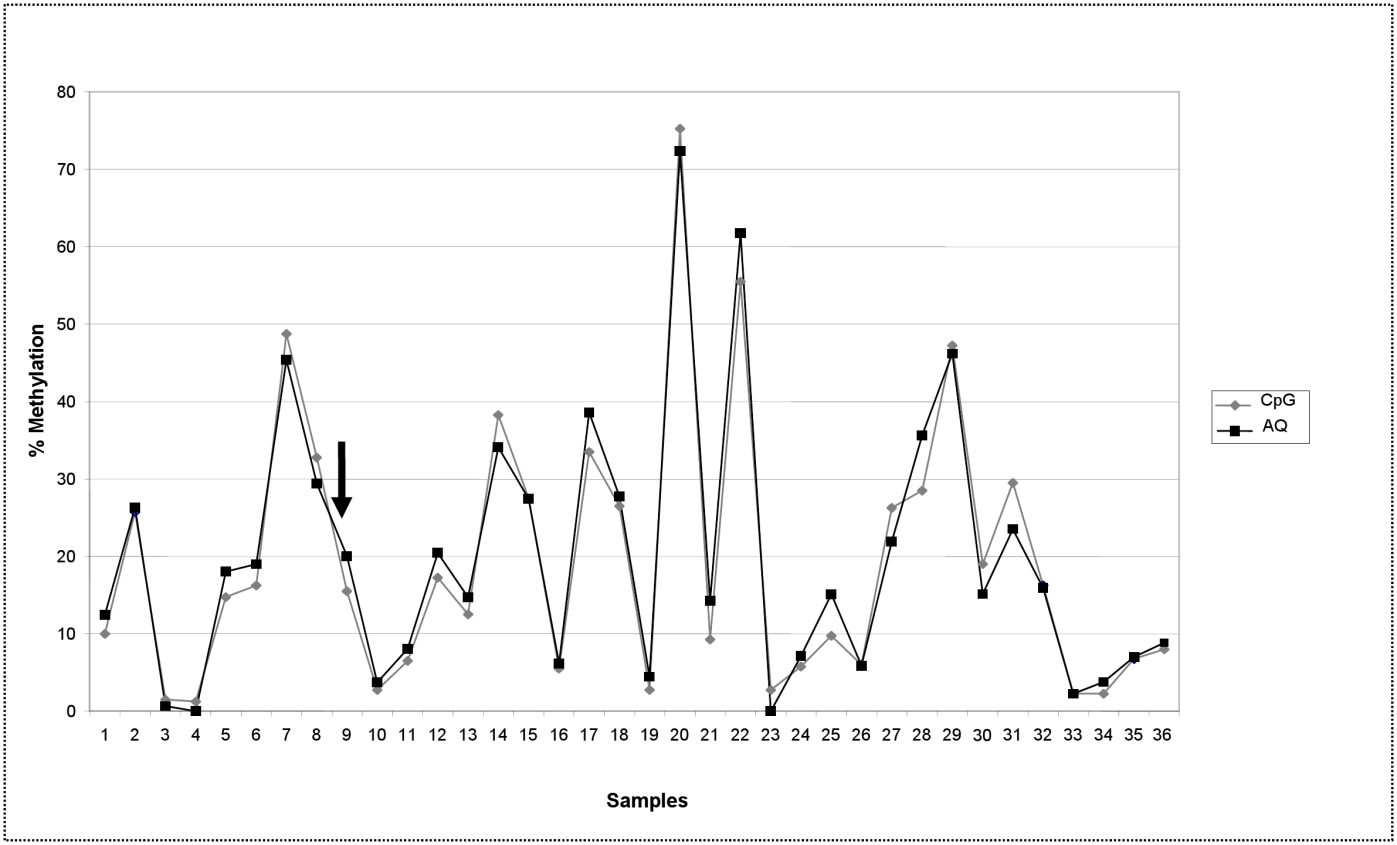
GSTP1 methylation analysis of DNA samples (PC study) tested in duplicate with PyroMark CpG mode and AQ mode. DNA samples (N = 36) obtained from paraffin embedded prostate tumor tissue of patients involved in “PC study” [24]. Mean methylation percentage of 4 CpGs in the promoter of GSTP1 gene is reported. Target sequence: 72 bp of GSTP1 promoter (Gene Bank M24485 at position 1001–1072); CpGs at position 1038, 1040, 1043, 1049. Deviation between paired samples ranged from 0 to 7.15%. One red score was assigned by the CpG mode to the sample indicated by the arrow. No sample has a red score assigned by the AQ mode.

Fig 1 shows the results obtained by testing the “PC study” DNA sample series (N = 36) for GSTP1 promoter methylation. Methylation analysis was performed for all the samples in duplicate using the same preliminary PCR products and pyrosequencing profiles, primers included. Analysis was conducted onto both PyroMark Q96 and a Q24 system, which harbor the software for quantification in AQ and CpG mode respectively, and which provide their own software-based quality assessment of the results. Average of methylation percentage of the 4 CpGs investigated in the gene promoter was reported. Deviation between paired samples analyzed with the two “modes” ranged from 0 to 7.15%. Only one red score warning of inadequate quality of the result was assigned by the Q24 software in CpG mode and no red scores were assigned by the Q96 software in AQ mode. The agreement computed with a threshold limit of 5% of methylation to distinguish unmethylated from methylated samples was excellent (*k* = 1).

Plot according to Bland-Altman (S1A Fig) shows that the two methods gave on average similar values (mean difference: -0.66, 95% CI: -1.7 to 0.4) and that the limits of agreement were not too wide (-7.0 and 5.7).

### MGMT methylation analysis employing PyroMark CpG and AQ mode

**Fig 2:**
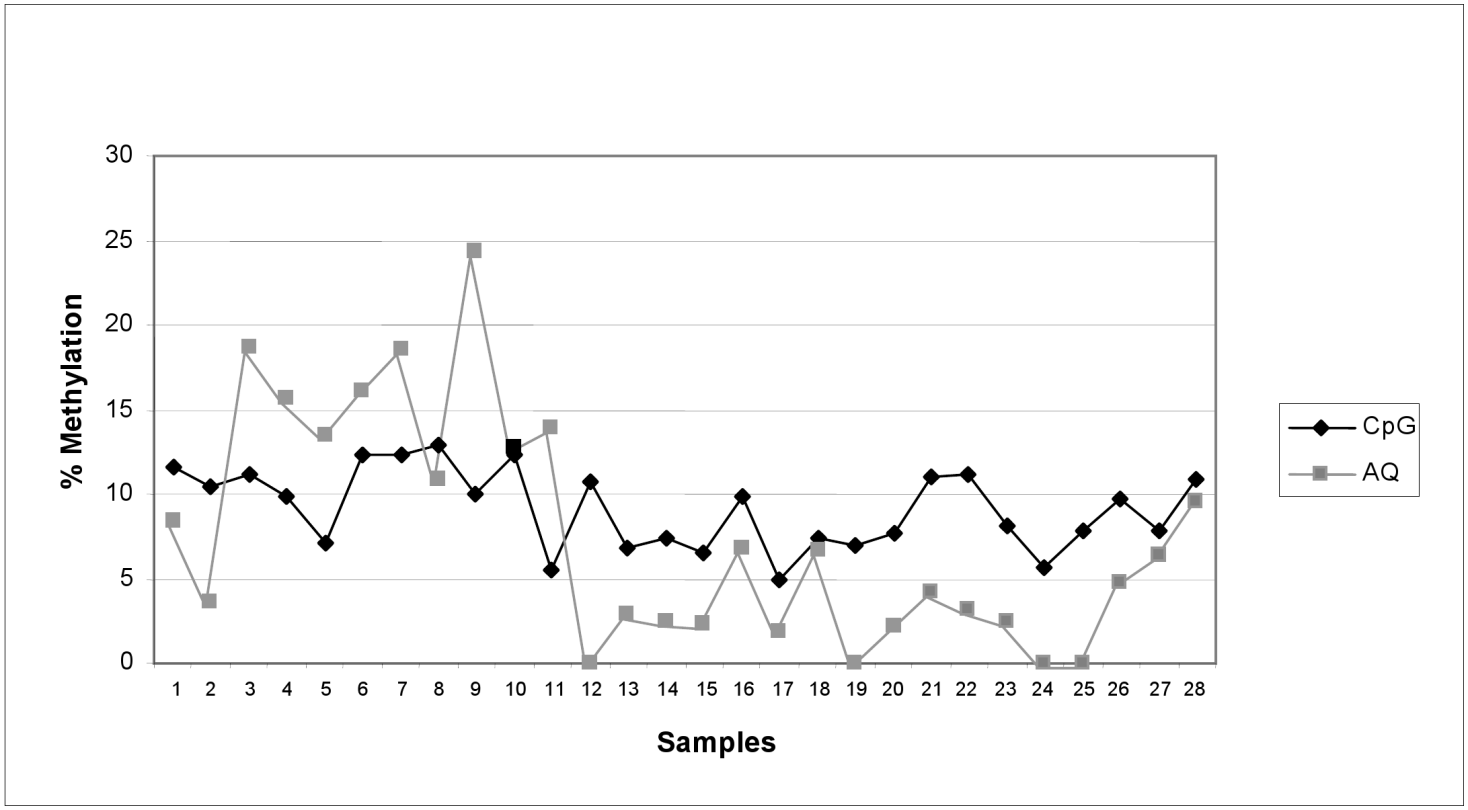
MGMT methylation analysis of DNA samples (DD study) tested in duplicate onto PyroMark CpG mode and AQ mode. DNA samples (N = 28) obtained buffy coats of patients involved in “DD study” [25]. Mean methylation percentage of 6 CpGs in the promoter of MGMT gene is reported. Target sequence: 194 bp of MGMT (Gene ID 4255 at position 44526–44719); CpGs at position 44600, 44604, 44607, 44614, 44621, 44623. Deviation between paired samples ranged from 0.3 to 14.4%. The CpG mode assigned 14 red score. No sample has a red score assigned by the AQ mode.

Fig 2 shows paired results obtained by testing the “DD study” DNA sample series (N = 28) for MGMT promoter methylation. Methylation analysis was performed for all the samples with the same approach described for GSTP1. We observed deviation between replicates analyzed with the two modes ranging between 0 and 14.4%. The concordance obtained by computing data with the threshold limit of 5% of methylation to distinguish unmethylated from methylated samples was poor (negative *k* agreement). The CpG mode generated 14/28 (50%) red scores, the AQ mode none. Paired samples receiving a red score with CpG mode had a yellow or even a blue score when using the AQ mode.

Plot according to Bland-Altman (S1B Fig) shows that analyses on MGMT with the two modes revealed wide limits of agreement (-10.5 to 13.7) and a negative correlation between differences and means (p = 0.003).

### LINE-1 methylation analysis employing PyroMark CpG and AQ mode

**Fig 3:**
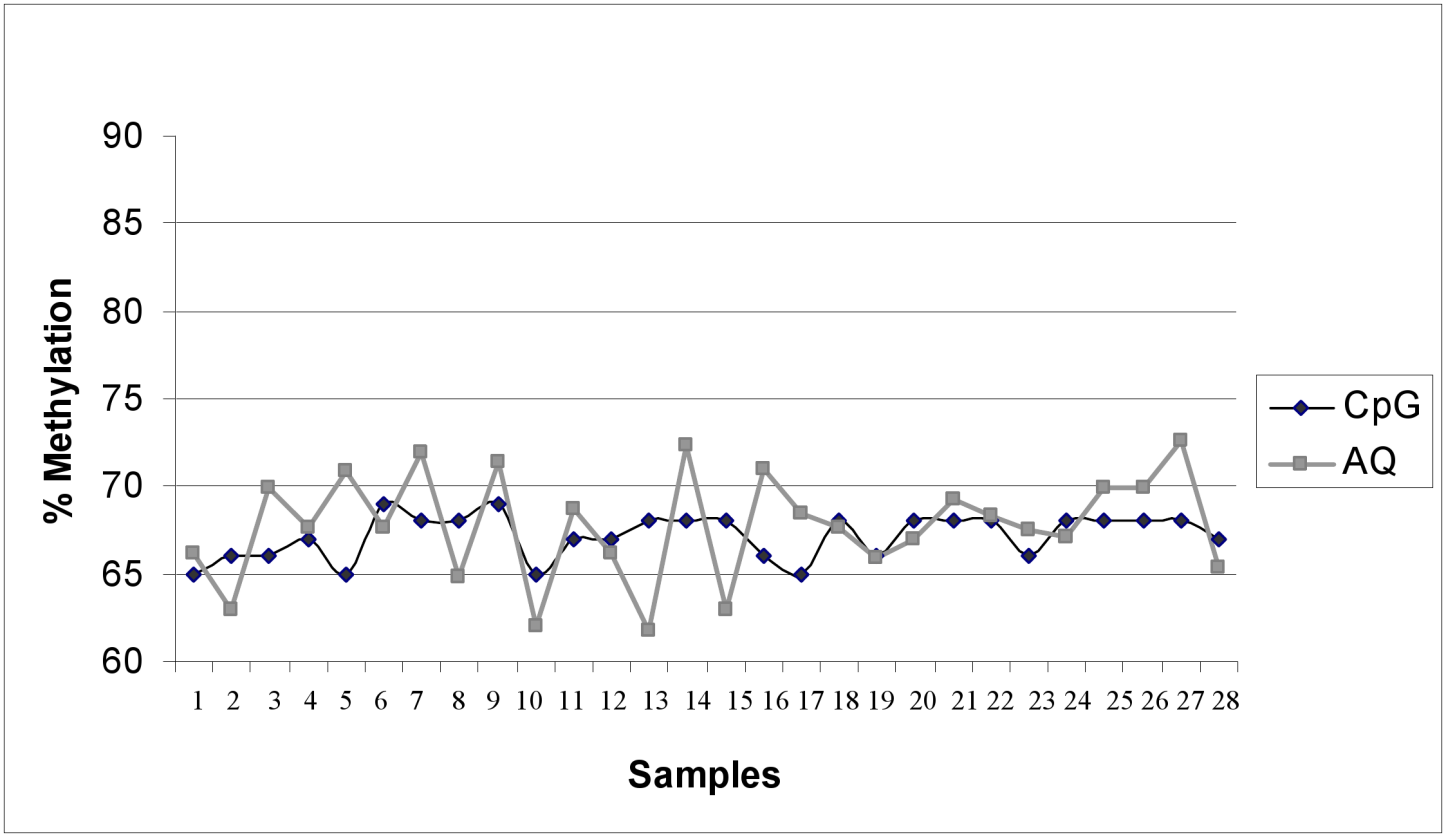
LINE-1 methylation analysis of DNA samples (DD study) tested in duplicate onto. PyroMark CpG mode and AQ mode. DNA samples (N = 28) obtained from buffy coats of patients involved in “DD study” [25]. Mean methylation percentage of 6 CpGs in the promoter of LINE-1 gene is reported. Target sequence: 108 bp of LINE-1 (GeneBank X58075.1 at position 117–224); CpGs at position 156, 131, 165, 167, 172, 182. Deviation between paired samples ranged from 0.5 to 4.2%. No red scores were assigned by the AQ mode. CpG mode assigned blue score only to the first CpG of each sample: all the other CpG in each samples received red or yellow scores.

Fig 3 shows paired results obtained by testing the “DD study” DNA sample series (N = 28) for LINE-1 promoter methylation of a template sequence including 6 CpGs. The same methodological approach described above was used. We obtained a mean percentage of methylation of 70%. Deviation between replicates ranged from 0.5 to 4.2%.

The AQ mode always assigned a blue score to all the 6 investigated CpGs (S2A Fig). Conversely, the CpG mode assigned a blue score only to the first investigated CpG in all the samples (S2B Fig). All the other CpGs in all the samples received a yellow (42% of all tested CpGs) or a red (57% of all tested CpGs) score.

Plot according to Bland-Altman (S1C Fig) revealed in the methylation analyses on LINE-1 with the two modes a mean difference of 1.0 (95% CI: 0.18 to 1.8). The limits of agreement (-3.2 to 5.2) have to be considered wide taking into account the small biological variability in LINE-1 methylation levels. A negative correlation between differences and means in LINE-1 methylation levels between the two modes is present (p value from the Pitman’s test = 0.006).

## Discussion

Pyrosequencing has emerged as an alternative method of nucleic acid sequencing, well suited for many applications which aim to characterize small DNA target sequences, whereas other available systems are more commonly used for global DNA analysis approaches (e.g.: microarray-based-Genome Wide Analysis; 454 array-based pyrosequencing platform, Life Sciences—Roche Diagnostics, Bonn, Germany, Illumina, San Diego, California; MALDI-TOF Mass Spectrometry, AB SCIEX Framingham, MA). Single Nucleotide Polymorphisms (SNPs), mutations, bacteria and viral types, sequences from cDNA library and methylated CpG sites can be efficiently investigated by pyrosequencing [10, 11].

For these applications, and also for DNA methylation analysis, published results have been widely obtained also recently by using software for analysis in AQ mode onto the PyroMark Q96. The use of PyroMark Q24 as well as of software for analysis in CpG mode is more rarely reported [9, 30–35] although the latter was specifically generated for DNA methylation assessment. Proof of equivalent performance in CpG methylation analysis of the two software is not available, and we are not aware of comparison of methylation results from replicates tested with both software.

Our paired results obtained with the two “modes” in the GSTP1 methylation analysis were in agreement. The GSTP1 methylation assay was set up at its best performance, thus reliable results were obtained with both the analytical systems, supported by blue scores assigned by the two software. The GSTP1 promoter is usually poorly methylated in normal cells, as the gene is physiologically expressed, and methylation rate was expected in normal conditions nearby the assay detection limit [36]. Every increase in the methylation rate can be therefore interpreted as a modification of the physiological pathway potentially involved in the outcome under investigation. From this point of view the above reported range of deviation (0–7.15%) between replicates can be considered within the limits of tolerance, and does not impair the reliability of the results, nor the conclusions that can be drawn by the analyses.

Conversely, paired results obtained with the two “modes” in the MGMT methylation analysis were highly discordant. In these assays the deviation between replicates acquires relevance, because the two modes differently identified methylated and un-methylated samples, weakening the reliability of the results. While CpG mode highlighted warnings for the interpretation of these results, likely due to a PCR assay not set up at its best performance yet, the AQ mode passed many results as acceptable with potential impact on the reliability of the final association of methylation events with the outcome.

When methylation average differences are supposed to be small among samples of a study series, accuracy in measuring quantitative results becomes more relevant to support evidence of associations. Indeed, methylation levels of intersperses sequences (e.g. LINEs) have been described to be slightly different (<5–10%) between pathological and physiological conditions [37,38]. LINE sequences are physiologically methylated and are commonly investigated as surrogates of global hypomethylation events. We tested healthy subjects and obtained a mean percentage of methylation of 70%, consistent with the blood detection average reported in the literature (ranges 66%-82.5% [39,40], 71.9% [41], 73.1% [42], 70–76% [26]). The *k* agreement was not assessed as meaningless in this context where, by testing hypomethylation events, we could only obtain less positive but unlikely negative (i.e. unmethylated) samples. The deviation between replicates (0.5–4.2%) that we obtained with the two modes could be misleadingly considered limited. The small biological variability in LINE-1 methylation levels has to be taken into account as it may potentially underline alterations. The score assignment by the two modes was very different: a more permissive performance of AQ mode is in contrast with the strong warnings of CpG mode on the need to improve PCR and pyrosequencing efficiency, although the assay profile as well as the primer sequences were chosen according to the literature [26]. If quantization is performed in AQ mode, no alert for potential unreliability of the results would emerge and risk of wrong classification and incorrect conclusions could not be excluded.

Both for LINE-1 and MGMT the negative correlation from the Bland-Altman plots translated into a qualitative change: CpG mode gave higher values for low methylation levels and lower values than AQ mode for higher methylation levels.

The discrepancy in quality score assignment by the two software modes lies on the different criteria which represent the stringency level (SL) for the assay: these levels (“low”, “normal”, “high” SL) are not comparable between AQ and CpG mode. Also if a low SL was manually set for CpG mode in the aforementioned assay, we would be unable to get acceptable scores for all CpGs: even the low SL of the CpG mode appeared more stringent than the high SL of the AQ mode.

In addition, default acceptable minimum height of single peaks in the pyrogram is quite different in the two modes (CpG: 20 RLU; AQ: none fixed limit), leading to a more stringent identification of methylation signals from background noise in the CpG mode.

## Conclusions

This is, at our knowledge, the first study comparing the performance in quantification of CpG methylation of the two mode analyses provided by PyroMark systems. Our data show discrepancy in the pyrosequencing software-based quality assignment of methylation results between the two mode of analyses. The less permissive criteria included into the Pyro Q-CpG^™^ software for quantification in CpG mode make explicit unsatisfactory performance of the assays for quantitative evaluation of DNA methylation at CpG sites. Therefore, the CpG mode of analysis ensures a better accuracy in the quantification of DNA methylation compared with the widespread used AQ mode. The potential drawbacks emerged in test accuracy could impact on the reliability of the results, and lead to consider AQ mode as not properly adequate for quantification of CpG methylation. Upgraded software versions which include the CpG mode are available for both Q24 and Q96 instrument. Basing on our data this implementation, in agreement with previous suggestions [13], is strongly advisable to improve the quality of the CpG methylation analysis results achievable by pyrosequencing.

## Supporting Information

S1 Fig (A-B-C). Methylation results plots according to Bland-Altman and Pitman’s Test of difference in variance. Comparison of the two methylation measurements obtained in AQ mode and in CpG mode by plots to evaluate difference in variance.
(TIF)

S2 Fig (A-B). Summary of the analysis of LINE-1 methylation on DNA samples in DD study. Comparison in replicates of the colour score assignment with the two software.
(TIF)
