# Supplementary figures and images for: Performance of Different Analytical Software Packages in Quantification of DNA Methylation by Pyrosequencing
Source: PLoS One. 2016 Mar 2;11(3):e0150483. doi: 10.1371/journal.pone.0150483 (PMC4775062; doi:10.1371/journal.pone.0150483)

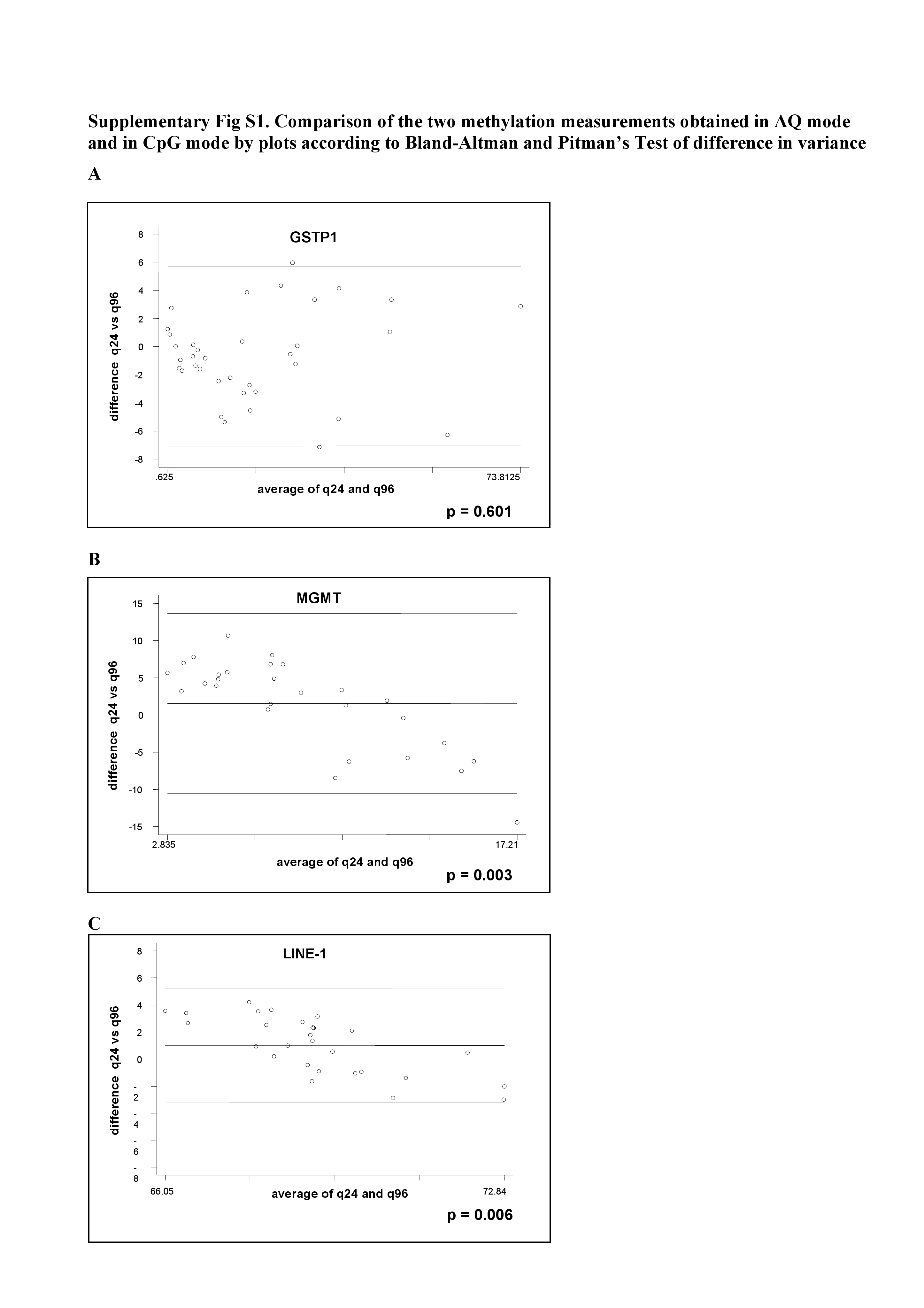

Supplement: S1 Fig — Comparison of the two methylation measurements obtained in AQ mode and in CpG mode by plots to evaluate difference in variance. (TIF) [file pone.0150483.s001.tif]

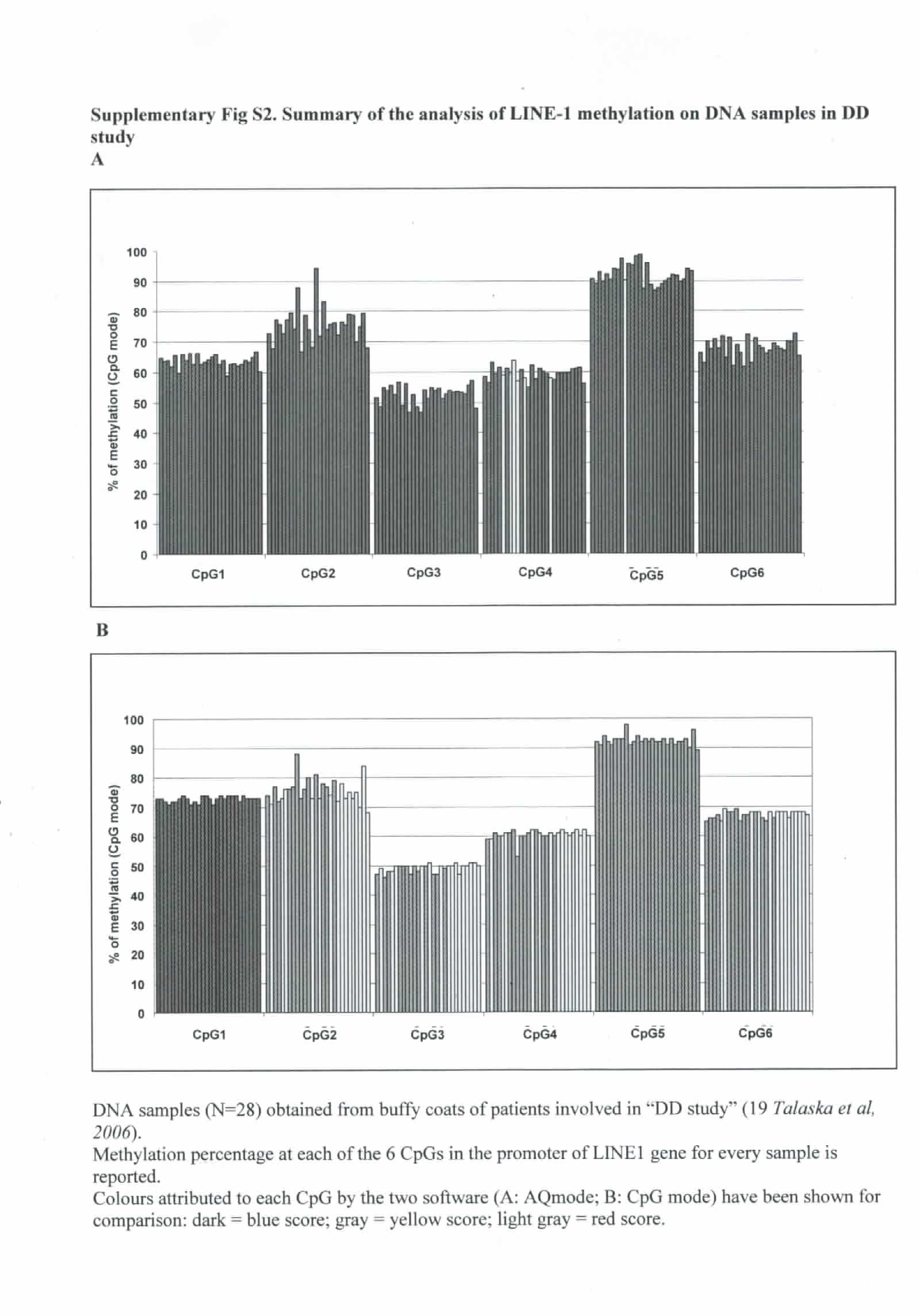

Supplement: S2 Fig — Comparison in replicates of the colour score assignment with the two software. (TIF) [file pone.0150483.s002.tif]
